# Including a Lower-Extremity Component during Hand-Arm Bimanual Intensive Training does not Attenuate Improvements of the Upper Extremities: A Retrospective Study of Randomized Trials

**DOI:** 10.3389/fneur.2017.00495

**Published:** 2017-09-26

**Authors:** Geoffroy Saussez, Marina B. Brandão, Andrew M. Gordon, Yannick Bleyenheuft

**Affiliations:** ^1^Institute of Neuroscience, Université catholique de Louvain, Brussels, Belgium; ^2^Universidade Federal de Minas Gerais, Belo Horizonte, Brazil; ^3^Department of Biobehavioral Sciences, Teachers College, Columbia University, New York, United States

**Keywords:** cerebral palsy, hemiplegia, intensive intervention, upper extremity, lower extremity, bimanual training, motor function, interlimb coordination

## Abstract

Hand-Arm Bimanual Intensive Therapy (HABIT) promotes hand function using intensive practice of bimanual functional and play tasks. This intervention has shown to be efficacious to improve upper-extremity (UE) function in children with unilateral spastic cerebral palsy (USCP). In addition to UE function deficits, lower-extremity (LE) function and UE–LE coordination are also impaired in children with USCP. Recently, a new intervention has been introduced in which the LE is simultaneously engaged during HABIT (Hand-Arm Bimanual Intensive Therapy Including Lower Extremities; HABIT-ILE). Positive effects of this therapy have been demonstrated for both the UE and LE function in children with USCP. However, it is unknown whether the addition of this constant LE component during a bimanual intensive therapy attenuates UE improvements observed in children with USCP. This retrospective study, based on multiple randomized protocols, aims to compare the UE function improvements in children with USCP after HABIT or HABIT-ILE. This study included 86 children with USCP who received 90 h of either HABIT (*n* = 42) or HABIT-ILE (*n* = 44) as participants in previous studies. Children were assessed before, after, and 4–6 months after intervention. Primary outcomes were the ABILHAND-Kids and the Assisting Hand Assessment. Secondary measures included the Jebsen-Taylor Test of Hand Function, the Pediatric Evaluation of Disability Inventory [(PEDI); only the self-care functional ability domain] and the Canadian Occupational Performance Measure (COPM). Data analysis was performed using two-way repeated-measures analysis of variance with repeated measures on test sessions. Both groups showed similar, significant improvements for all tests (test session effect *p* < 0.001; group × test session interaction *p* > 0.05) except the PEDI and COPM. Larger improvements on these tests were found for the HABIT-ILE group (test session effect *p* < 0.001; group × test session interaction *p* < 0.05). These larger improvements may be explained by the constant simultaneous UE–LE engagement observed during the HABIT-ILE intervention since many daily living activities included in the PEDI and the COPM goals involve the LE and, more specifically, UE–LE coordination. We conclude that UE improvements in children with USCP are not attenuated by simultaneous UE–LE engagement during intensive intervention. In addition, systematic LE engagement during bimanual intensive intervention (HABIT-ILE) leads to larger functional improvements in activities of daily living involving the LE.

## Introduction

Cerebral palsy (CP) is the most common cause of pediatric motor disability with a prevalence ranging from 2 to 3.6 out of 1,000 children in western countries (1, 2). Motor disorders are often accompanied by sensation, perception, cognition, behavior, communication, and epilepsy disorders (1). Although the lesions are established from birth and are non-progressive, the motor impairments experienced by children with CP affect their autonomy and functional outcomes during their life-span. Moreover, motor symptoms such as impaired ability to walk may worsen during development (3).

One of the most disabling long-term functional deficits in children with unilateral spastic cerebral palsy (USCP) is impaired manual dexterity, i.e., impaired skilled hand movements and precision grip abilities (4). Upper-extremity (UE) impairments may affect functional independence, especially for activities of daily living requiring bimanual coordination (e.g., buttoning one’s shirt). It is now well known that intensive interventions based on motor skill learning principles and goal-directed training are effective for improving UE function in children with USCP (5). Constraint-Induced Movement Therapy (CIMT) was the first intensive intervention adapted to children with USCP (6). CIMT was first designed for adults with stroke and subsequently adapted to children with USCP showing improvements in hand function (5). Taking advantage of the key ingredient of CIMT (intensive practice with the affected UE), Charles and Gordon developed an alternative intensive bimanual approach termed “Hand-Arm Bimanual Intensive Therapy” (HABIT) (7). HABIT was developed with recognition that the combined use of both hands was necessary to increase functional independence in children with USCP (7). Focusing on improving bimanual coordination through structured play and functional activities during HABIT demonstrated efficacy to improve UE function in children with USCP (5).

Both HABIT and CIMT focus only on the UE of children with USCP. Though the lower extremity (LE) is generally less affected than UE in children with USCP, impairments observed in the affected LE range from an isolated equine ankle to hip flexion and adduction with a fixed knee (8). Children with USCP are then unable to achieve postural symmetry while standing, systematically presenting with an overload on one bodyside (8). They also frequently encounter limitations in walking abilities (3). Besides the LE impairments, UE–LE coordination is often impaired in children with USCP (9, 10). This coordination is frequently used in daily living activities (e.g., walking while carrying an object in the hand, climbing stairs while using the railing). A program that simultaneously trains the UE and LE in children with USCP is thus of interest since the UE impairments in children with CP remain stable through time (11) while walking and other LE abilities may decline during development (3). In 2014, taking advantage of the key ingredients in HABIT (intensive bimanual practice), Bleyenheuft and Gordon developed a new intervention focusing on both the UE and LE entitled “Hand-Arm Bimanual Intensive Therapy Including Lower Extremities” (HABIT-ILE) (12). Positive effects of this therapy focusing on both the UE and LE through structured play and functional activities have been demonstrated both for the UE and the LE of children with USCP (13) as well as, more recently, for children with bilateral CP (14). However, it is unknown whether the introduction of a systematic LE engagement in addition to a bimanual intervention may lead to attenuated improvements in UE compared to traditional HABIT due to shifts in attention (multitasking). This retrospective study aimed to compare changes in the UE of children with USCP undergoing 90 h of intensive bimanual intervention either with (HABIT-ILE) or without (HABIT) a LE component. We hypothesized that the introduction of systematic LE training simultaneously added to the bimanual training would lead to reduced improvements in the UE during HABIT-ILE compared to traditional HABIT.

## Materials and Methods

### Participants

This project was conducted at the Motor Skill Learning and Intensive Neurorehabilitation (MSL-IN) lab from the Université catholique de Louvain (UCL), Brussels, Belgium and the Center for CP Research (CCPR), Teachers College, Columbia University, New York, USA with approval by the ethical committees of the respective universities.

Data were collected from 86 children with USCP who took part in HABIT (*n* = 42; CCPR, Columbia University) or HABIT-ILE (*n* = 44; MSL-IN lab, UCL) intervention between 2010 and 2015 as part of other trials. Common supervisors were present in both sites. Fifteen children were involved in a RCT in Brussels, Université catholique de Louvain, investigating the effect of HABIT-ILE intervention in children with USCP (13) and 25 in another RCT in New York, Columbia University, aiming to compare the effect of intensive bimanual training with and without structured progression of skill difficulty (15). The data of the other 46 children are included in RCTs currently under way (Clinical Trials identifiers: NCT02667613 and NCT02667613).

Children in both groups had a MACS level between I and III (16). Their clinical characteristics are shown in Table 1.

**Table 1:** General characteristics.

	HABIT	HABIT-ILE	*p*-Value
	(*n* = 42)	(*n* = 44)	*T-*test
**General characteristics**			

Gender			
Male	23	18	–
Female	19	26	–
Age: years⋅months	8.85 ± 3.09	8.79 ± 2.17	0.915
Hemiparetic side			
Right: *n* (%)	30 (71.43)	26 (59.09)	
Left: *n* (%)	12 (28.57)	18 (40.91)	
MACS			0.195
1	10	9	–
2	21	33	–
3	11	2	–
4	0	0	–

**Baseline assessment**			

ABILHAND-kids			
Subjects (*n*)	41	42	–
Score (logits)	1.66 ± 1.27	2.18 ± 1.60	0.104
AHA			
Subjects (*n*)	39	42	–
Score (% of logits)	59.84 ± 10.39	63.23 ± 15.52	0.255
JTTHF-MA hand			
Subjects (*n*)	41	34	–
Score (seconds)	343.82 ± 271.78	373.39 ± 273.49	0.641
PEDI			
Subjects (*n*)	41	40	–
Raw score (/73)	64.31 ± 7.06	60.72 ± 7.85	0.033*
COPM performance			
Subjects (*n*)	40	32	–
Raw score (/10)	3.26 ± 1.46	3.48 ± 1.16	0.491
COPM satisfaction			
Subjects (*n*)	40	32	–
Raw score (/10)	4.07 ± 2.25	3.88 ± 1.37	0.680

*MACS, Manual Ability Classification System; JTTHF, Jebsen-Taylor Test of Hand Function; AHA, Assisting Hand Assessment; PEDI, Pediatric Evaluation of Disability Inventory (self-care functional ability domain); COPM, Canadian Occupational Performance Measure; MA, more-affected; LA, less affected; HABIT, Hand-Arm Bimanual Intensive Therapy; HABIT-ILE, Hand-Arm Bimanual Intensive Therapy Including Lower Extremities*.

*All quantitative variables from baseline assessment are presented with mean ± SD for each group*.

*p < 0.05.

Consistent with previous HABIT and HABIT-ILE trials (13, 17), inclusion criteria were: (1) aged between 5 and 18 years, (2) ability to grasp light objects and lift the more-affected arm 15 cm above a table surface, (3) ability to follow instructions and complete testing. Exclusion criteria were: (1) uncontrolled seizures, (2) orthopedic surgery or botulinum toxin injections less than 12 months before or within the study period, (3) possibility of treatment/testing interference because of visual problems. Participants and caregivers provided informed consent.

### Interventions

Both HABIT and HABIT-ILE are intensive interventions developed for children with USCP (7, 12). Basic motor skill learning principles are applied in these two interventions (18) with a structured practice of bimanual hand use induced through functional activities gradually increasing in complexity. These therapies are provided in a child-friendly context taking into account children’s and parent’s goals. They are provided in small groups (<12 children) using a 90-h camp model with intervention provided by at least one trained interventionist per child. Both interventions have demonstrated positive effects on UE function (and LE function for HABIT-ILE) in children with USCP (5, 13). Intensity is provided using a high dosage of intervention but also through a high motor engagement time including voluntary controlled (non-guided) movements. During both interventions, tasks are graded and the environment is adapted to allow success of the child but still challenging the motor demands. Children received instructions from the interventionist but also engaged in active problem solving. Individual therapy goals and strategies are reevaluated during daily team meeting with supervisors providing consultation to the interventionists.

During HABIT, children practiced bimanual functional activities 6 h a day for 15 consecutive weekdays (90 h) (19).

During HABIT-ILE, children were involved in bimanual activities with simultaneous engagement of the LE and postural control during play and functional activities 9 h a day for 10 consecutive weekdays (90 h) (12).

Except for the differences in dosing schedule and the LE component, the therapeutic principles used in these interventions are identical. All table activities were identical except that children in HABIT-ILE were either seated on a fitness ball, standing, or standing on a balance platform. Furthermore, common supervisors were in charge in both sites to ensure congruence in therapeutic directions.

### Assessments Procedure

Assessments were performed at pre-camp (T1), after 90 h of therapy (T2), and at follow-up (T3; 4.5–6 months later) (13, 17). Each child was tested individually in a quiet room by a physical or occupational therapist after being informed of test procedures.

Two questionnaires [Pediatric Evaluation of Disability Inventory (PEDI) and ABILHAND-Kids], a videotaped assessment [Assisting Hand Assessment (AHA)], one motor test [Jebsen-Taylor Test of Hand Function (JTTHF)], and a measure of the functional goals performance and satisfaction [Canadian Occupational Performance Measure (COPM)] were performed at each test session. The primary outcomes are the ABILHAND-Kids and the AHA.

#### Primary Outcomes

The ABILHAND-Kids (20) is a reliable and valid questionnaire measuring children’s manual ability. The child’s difficulty in performing each activity is scored by the parents using a 3-level response scale (impossible/difficult/easy). The total score based on 21 activities is then converted into a linear measure of manual ability (logits). This test was specifically developed for children with CP using the Rasch measurement model and showed a good reliability and reproducibility over time (20).

The aim of the AHA (21) is to measure the effectiveness with which the child makes use of his/her more-affected hand in everyday bimanual activities. Containing a set of normally bimanually handled selected toys, this video recorded test is conducted as a semi-structured play session lasting ~15–20 min. The 22 items of the AHA are rated on a four-point rating scale according to a manual with specific criteria describing each item and each step of the rating scale. Reliability and validity of this Rasch-built assessment has been proved for children from 18 months to 12 years old (22). The total score converted into linear measures (% of logits—AHA units) was blindly rated by a certified examiner from videotaped sessions.

#### Secondary Outcomes

The purpose of the JTTHF is to assess the UE function in simulated activities of daily living (23). Six subtasks were tested here: turning cards, lifting small objects, simulated feeding, stacking checkers, and picking up light and heavy cans. The writing task was excluded and the maximum time limit for each subtask was 180 s (24). Each hand was tested separately beginning with the less-affected hand and the child was asked to perform the tasks as fast as possible. The score (in seconds) is the time needed to perform all the tasks. The test–retest reliability of the JTTHF has been recently demonstrated in typically developing children aged from 6 to 10 years old (25) and normative values have been established for typically developing children (24, 26).

The PEDI (27, 28) is a questionnaire dedicated to the measurement of children’s performance and functional skills during daily activities. Only the self-care subscale functional ability domain was used here. Each of the 73 skills is rated by the parents as 0 (unable or limited to perform the skill) or 1 (usually able to perform the skill). The test score is the addition of the skills ratings. The PEDI has a good validity and intra-rater reliability (27, 29).

The COPM (30) is a measure of a client self-perception of occupational performance in the areas of self-care, productivity, and leisure. The COPM is administered using a semi-structured interview in which the children and/or his/her parent identify significant issues in daily activities, which are causing difficulty. The interview consists in four steps and focuses on activities that the child/parents want, need, or expect to perform. (1) The child/parents identify problems in occupational performance that are important and relevant and (2) determine priorities by rating the importance of each activities on a 10-point scale (from 1 “not important at all” to 10 “extremely important”). (3) The child/parents identify then the five most important problems they perceive during daily activities. (4) Each of the five most important problems is then rated on a 10-point scale regarding to the child’s performance (1 “not able to do it at all,” 10 “able to perform extremely well”) and parents’ satisfaction (1 “not at all satisfied,” 10 “extremely satisfied”). Mean scores can be calculated for performance and satisfaction. The COPM was then used to predefine and establish the functional objectives for the HABIT and HABIT-ILE protocols. Validity and reproducibility have been shown for the COPM (31, 32).

### Statistics

Statistical analyses were performed using the software Sigmastat 3.5. Two tailed *t*-tests were performed to compare the two groups at baseline assessment. A 2 (groups) × 3 (test sessions) analysis of variance (ANOVA) with repeated measures on test sessions was used to compare both groups. The six statistical assumptions for running ANOVAS (continuous aspect of dependent variable, independence of the two variables, independence of observations, no significant outliers, approximately normal distribution, and homogeneity of variance) were tested in every outcome. Although normality was not systematically observed in all subgroups, ANOVAs were used since recent research in statistics demonstrated that ANOVA is a robust test against the normality assumption (33). All other assumptions were systematically met. Homogeneity of variance was tested and verified using the Fisher test and the Howell’s procedure (34). *Post hoc* follow-up tests were systematically performed where a main effect or a significant interaction was observed using the Newman–Keuls *post hoc* including an adjustment for multiple comparisons (adjusted *p*-values). In addition, the number of children reaching a clinical significance of change is reported using the empirical rule of effect size (ERES; change of each child considered as clinically meaningful if >0.5 SD of the whole sample at baseline) (35). Significance level was set at 0.05.

## Results

Of the 86 participants, 44 received HABIT-ILE and 42 received HABIT. For the HABIT-ILE group, 7 children included in 2011 did not have JTTHF or COPM measurements since these tests were not performed in the assessment battery that year. All outcomes were similar at baseline, except the PEDI (secondary outcome) (Table 1). Children with a missing value at one of the assessment sessions were excluded for the analysis of this variable (Table 2). Two kids in the HABIT-ILE group did not show up for a follow-up assessment and were then excluded from all the analysis. Mean values at different test sessions, as well as statistics and number of subjects with significant changes are reported in Table 2.

**Table 2:** Upper-extremity changes.

				2-way RM ANOVA (2 groups × 3 test sessions)						Clinical significance of change (ERES)	
					
	Pre-camp	Post-camp	Follow-up	Testing session			Interaction			Significant change if >0.5SD *n* = subjects with significant change	
						
	Mean ± SD	Mean ± SD	Mean ± SD	df	*p*-Value	*F*-value	df	*p*-Value	*F*-value	Pre vs post	Post vs follow-up
**Primary outcomes**											

ABILHAND-Kids (logits)				df = 2	*p* < 0.001*	*F* = 51.19	df = 2	*p* = 0.138	*F* = 2.00		
HABIT (*n* = 41)	1.66 ± 1.27	2.38 ± 1.46	2.73 ± 1.42	Pre ≠ post, follow-up						22 out 41 (53%)	12 out 41 (29%)
HABIT-ILE (*n* = 42)	2.18 ± 1.60	3.41 ± 1.72	3.52 ± 1.79	Pre ≠ post, follow-up						25 out 42 (59%)	8 out 42 (19%)
AHA (% of logits)				df = 2	*p* < 0.001*	*F* = 19.48	df = 2	*p* = 0.130	*F* = 2.06		
HABIT (*n* = 39)	59.84 ± 10.39	62.66 ± 11.34	61.53 ± 10.31	Pre ≠ post, follow-up						10 out 39 (25%)	2 out 39 (5%)
HABIT-ILE (*n* = 42)	63.23 ± 15.52	67.19 ± 15.23	67.3 ± 15.79	Pre ≠ post, follow-up						7 out 42 (16%)	7 out 42 (16%)

**Secondary outcomes**											

JTTHF-MA hand (sec)				df = 2	*p* < 0.001*	*F* = 23.83	df = 2	*p* = 0.230	*F* = 1.48		
HABIT (*n* = 41)	343.8 ± 271.7	274.8 ± 253.0	287.0 ± 264.8	Pre ≠ post, follow-up						8 out 41 (19%)	1 out 41 (2%)
HABIT-ILE (*n* = 34)	373.3 ± 273.4	314.5 ± 265.8	289.1 ± 221.4	Pre ≠ post, follow-up						5 out 34 (14%)	5 out 34 (14%)
PEDI (raw score)				df = 2	*p* < 0.001*	*F* = 60.54	df = 2	*p* = 0.027*	*F* = 3.68		
HABIT (*n* = 41)	64.3 ± 7.06	67.8 ± 6.56	68.5 ± 5.84	Pre ≠ post, follow-up						17 out 41 (41%)	7 out 41 (17%)
HABIT-ILE (*n* = 40)	60.7 ± 7.85	66.9 ± 5.99	67.2 ± 5.93	Pre ≠ post, follow-up						27 out 40 (67%)	12 out 40 (30%)
COPM perf (raw score)				df = 2	*p* < 0.001*	*F* = 260.04	df = 2	*p* = 0.049*	*F* = 3.08		
HABIT (*n* = 40)	3.26 ± 1.46	6.41 ± 1.55	6.42 ± 1.20	Pre ≠ post, follow-up						36 out 40 (90%)	11 out 40 (27%)
HABIT-ILE (*n* = 32)	3.48 ± 1.16	7.44 ± 1.22	7.35 ± 1.04	Pre ≠ post, follow-up						32 out 32 (100%)	7 out 32 (21%)
COPM sat (raw score)				df = 2	*p* < 0.001*	*F* = 164.63	df = 2	*p* = 0.071	*F* = 2.70		
HABIT (*n* = 40)	4.07 ± 2.25	7.35 ± 1.77	6.87 ± 1.28	Pre ≠ post, follow-up						31 out 40 (77%)	7 out 40 (17%)
HABIT-ILE (*n* = 32)	3.88 ± 1.37	7.97 ± 1.31	7.65 ± 1.30	Pre ≠ post, follow-up						31 out 32 (96%)	5 out 32 (15%)

*HABIT, Hand-Arm Bimanual Intensive Therapy; HABIT-ILE, Hand-Arm Bimanual Intensive Therapy Including Lower Extremities; AHA, Assisting Hand Assessment; JTTHF, Jebsen-Taylor Test of Hand Function; MA, more affected; LA, less affected; PEDI, Pediatric Evaluation of Disability Inventory; COPM, Canadian Occupational Performance Measure (performance and satisfaction measures); RM ANOVA, repeated-measures analysis of variance; ERES, empirical rule of effect size; df, degree of freedom*.

*p < 0.05.

### Primary Outcomes

**Figure 1:**
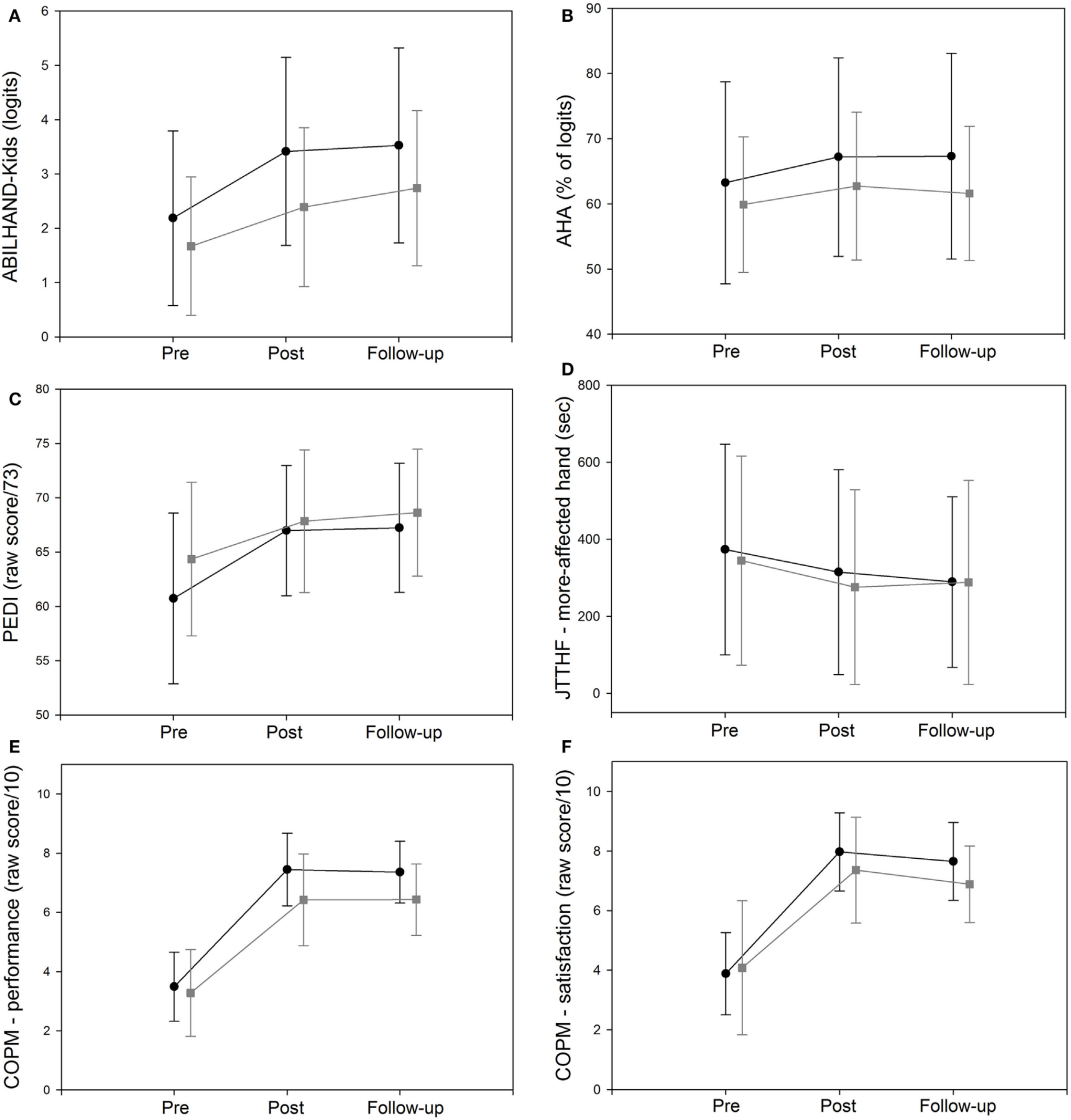
Mean ± SD of the mean (SD) score in HABIT and HABIT-ILE groups. 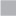 = HABIT; ● = HABIT-ILE for **(A)** the ABILHAND-Kids **(B)** the Assisting Hand Assessment (AHA), **(C)** the Pediatric Evaluation of Disability Inventory (PEDI) (self-care functional ability domain), **(D)** the Jebsen-Taylor Test of Hand Function (JTTHF) on the more-affected hand, **(E)** the Canadian Occupational Performance Measure (COPM) on performance measure, and **(F)** the COPM on satisfaction measure.

As illustrated in Figure 1, a significant improvement in the ABILHAND-Kids questionnaire was observed (main effect of test session *p* < 0.001) (Table 2). No significant group × test session interaction was found (*p* = 0.138) (Table 2).

For the AHA, as shown in Figure 1, both groups improved significantly (main effect of test session *p* < 0.001). No group × test session interaction was observed (*p* = 0.130; Table 2).

### Secondary Outcomes

Figure 1 shows the JTTHF results for the more-affected hand in the HABIT and HABIT-ILE groups. There was a significant effect of test session (*p* < 0.001) but no group × test session interaction was found (*p* = 0.230; Table 2).

For the PEDI, as illustrated in Figure 1, a significant test session effect was observed for both HABIT and HABIT-ILE groups (*p* < 0.001). A significant group × test session interaction (*p* = 0.027) was also found between the HABIT and HABIT-ILE groups (Table 2) showing larger improvements for the PEDI in the HABIT-ILE (+6.5) than in the HABIT (+4.268) group as confirmed by *post hoc* analysis (Table 2), with both groups remaining stable at follow-up.

As shown in Figure 1, there was a significant effect of test session (*p* < 0.001) for the COPM performance and satisfaction measures during both HABIT and HABIT-ILE interventions (Table 2). No group × test session interaction was found for the satisfaction measure (*p* = 0.071) but, for the performance measure, a significant group × test session interaction (*p* = 0.049) was found with the *post hoc* analysis showing that children from the HABIT-ILE group (+3.871) had a significantly larger improvement than those from the HABIT group (+3.154) (Table 2).

Canadian Occupational Performance Measure goals were analyzed by five experts and scored as involving the UE, LE, or the UE–LE coordination (Table 3). In both groups, around 60% of the goals involved solely the UE and at least one-third of the goals involved the UE–LE coordination. Only a few goals involved the LE alone.

**Table 3:** Upper and lower extremities implication in the Canadian Occupational Performance Measure goals.

	UE	LE	UE–LE
**HABIT**			
Goals (%)	59.04	0.53	40.43
**HABIT-ILE**			
Goals (%)	63.37	2.33	34.30

*UE, upper extremities; LE, lower extremities; UE–LE, upper and lower extremities*.

*Upper and lower extremities implication were determined by five experts who scored each of the HABIT and HABIT-ILE goals as focusing solely on the UE, the LE, or both the UE–LE in coordination. The goals were then categorized as focusing on the UE, LE, or UE–LE if at least three of the five experts answered in the same way (in case of disagreement, a sixth expert was asked to judge the UE, LE, and UE–LE implication)*.

### Clinical Significance of Change

Clinical significance of change was approached here using the ERES (Table 2).

For the ABILHAND-Kids questionnaire, the percentage of children presenting changes considered as clinically significant between pre and post-intervention testing sessions was 53 and 59% for the HABIT and HABIT-ILE groups, respectively, with 29 and 19% of changes reported as clinically significant between post-intervention and follow-up testing sessions. For the AHA, the reported percentage were 25 and 16% between pre and post-intervention testing sessions for the HABIT and HABIT-ILE groups, respectively, with 5 and 16% of changes reported as clinically significant between post-intervention and follow-up testing sessions.

For the secondary outcomes, changes in the HABIT and HABIT-ILE groups reported as clinically significant between pre and post-intervention testing sessions were 19 and 14% for the JTTHF, 41 and 67% for the PEDI, 90 and 100% for the COPM performance measure, and 77 and 96% for the COPM satisfaction measure.

## Discussion

The aim of this study was to compare changes in UE motor function of children with USCP following 90 h of intensive bimanual intervention either with (HABIT-ILE) or without (HABIT) a LE component. We hypothesized that the introduction of a systematic LE in addition to a bimanual intervention may lead to attenuated improvements in UE compared to regular HABIT. However, the results yielded similar UE motor improvements after both HABIT and HABIT-ILE for most assessments including our primary outcomes. While both groups showed significant improvements, larger improvements were observed for the PEDI and COPM performance measure for children of the HABIT-ILE group.

### Similar UE Improvements

It was hypothesized that, because of the addition of a LE component during HABIT-ILE, improvement in UE motor function could be attenuated in children with USCP. It has been demonstrated that children with USCP experience larger dual-task interference than typically developing children, both for cognitive (36) and motor dual tasks (10). Specifically, when performing a task in which they had to hold a box while walking, children with USCP demonstrated greater interference for the UE and LE than in typically developing controls (10). Considering UE–LE coordination during HABIT-ILE intervention as a complex process likely inducing dual-task interference, a decreased rate of learning in UE improvements was expected.

However, results showed significant improvements in both HABIT and HABIT-ILE groups for all assessment measures. The non-significant group × test session interactions observed for most of the tests means that both groups improved similarly in UE motor function. These findings can be explained as both HABIT and HABIT-ILE interventions were designed following the same methodological principles: 90 h of intensive bimanual structured practice with increasing motor difficulty and repetition of tasks during play and activities of daily living (7, 12). Although HABIT-ILE focuses on both UE and LE, there is still a constant use of the UE and engagement in bimanual coordination during the 90 h of therapy (12).

Previous studies showed that children participating in HABIT or HABIT-ILE were engaged in structured practice for 79–94 and 96% of the 90 h, respectively, of therapy time. The remaining time was spent in transitioning between tasks, choosing games, toileting (13, 15, 19). Concerning UE engagement, children spent on average 12–17% of the HABIT intervention time on part-practice (practicing a targeted movement while increasing repetition for 30 s using symmetrical or asymmetrical bimanual activities) and 83–88% on whole-practice tasks (e.g., card games, manipulative games, arts and crafts; performed for 15–20 min) (15, 19). HABIT-ILE consisted of an average of 21% of part-practice and 79% of whole-practice tasks for the UE (13). Using the same ingredients for the UE, our results suggest that the LE component does not compromise improvements in motor function of the UE.

#### Interpreting Clinical Significance of Change

Clinical significance of change was approached here using the ERES. For the ABIHAND-Kids questionnaire, the percentage of children presenting changes considered as clinically significant is congruent with previous randomized controlled trials in both interventions reporting significant improvements using the ABILHAND-Kids questionnaire and showing thus an effect on manual ability (13, 15). The lower percentage of children reaching a clinically significant improvement for the AHA may be linked to a moderate sensitivity to change of the test since some previous studies also highlighted significant improvements on the AHA with changes reported as below the smallest detectable difference threshold estimated of 5 AHA units (19, 37–39).

### Unexpected Significant Interactions

While both HABIT and HABIT-ILE groups showed significant improvements, a significant group × test session interaction was found between groups for the PEDI and the COPM performance measure, both showing larger improvements in the HABIT-ILE group.

For the PEDI, this might be explained by the fact that this questionnaire, in the self-care part used in this study, includes some items requiring the use of LE and postural control (e.g., body wash, dressing, use toilet), which are more likely to be improved in HABIT-ILE than in HABIT.

The importance of LE and especially of postural control for functional tasks has been highlighted by Domagalska et al., showing that the ability to gain independence at performing activities of daily living seemed to be determined by postural control abilities (8). LE impairments such as decreased range of motion during growth observed in children with USCP, may limit walking activities, which is referred as an essential activity for functional daily living activities (3). The slightly lower scores on the PEDI observed at baseline for the HABIT-ILE group could also have an effect (more room for improvement than in the HABIT group).

For the COPM, according to the literature, most of the functional goals defined as priority goals in children with CP, regardless age, focus on self-care (e.g., dressing, hygiene, toileting, bathing) (15, 40). Other functional goals frequently described as outcomes in children with USCP concerns mobility (e.g., transfers, transporting an object), play, and school activities (15, 40, 41). Moreover, at least one-third of the COPM goals defined in both groups were involving UE–LE coordination. While most goals are bimanual tasks (15) (>50% of the goals in our results), some of the priority COPM functional goals also require LE motor function, trunk control, and most importantly, UE–LE coordination.

In addition to their impairments in the UE and LE, children with USCP have impairments in the UE–LE coordination (9) and combined UE and LE motor tasks (e.g., walking with a box in the hands) are therefore also impaired (10). As UE–LE coordination is frequently used in functional daily living activities, the finding of impaired UE–LE coordination in children with USCP is in agreement with those showing at least one-third of the COPM items involving UE–LE coordination as well as self-care and mobility goals defined as priority functional goals on the COPM. Again, these goals including the UE–LE coordination are more likely to be improved when trained.

During HABIT-ILE, children spent around 54% of the intervention time sitting on a ball, 24% standing, 2% standing on a balance board, and 20% walking/running or jumping while manipulating objects using both hands (13). This intervention focusing on bimanual activities with continuous LE engagement has shown positive effects on both UE and LE (13). It seems thus consistent to find larger changes in the PEDI and COPM performance measure including a LE component than during HABIT alone.

This study demonstrated that in HABIT-ILE, UE improvements are similar to those obtained in HABIT for children with USCP. Until recently, HABIT and HABIT-ILE interventions have only been studied and provided to children with USCP. Regarding the improvements observed for these children, it seemed relevant to investigate whether similar improvements could be observed in children with bilateral CP (e.g., diplegia, quadriplegia) who are lacking evidence-based interventions (5). In 2017, the efficacy of HABIT-ILE in children with bilateral CP (no cognitive impairment, mainly GMFCS III) was examined in a quasi-randomized trial and showed significant improvements in both the UE and LE (14). Whether this UE–LE training can be transferred to children with cognitive impairment or with larger motor deficits (GMFCS IV to V) is not known and should be the focus of future investigations.

### Limitations

One limitation is that this study was not a randomized controlled trial. While children in both groups participated in different RCTs, the present comparison may have been affected from differences in the protocols between the two sites. However, it should be noted that there were always common supervisors present in both sites to standardize protocols.

Second, while both interventions were delivered over 90 h, HABIT and HABIT-ILE therapies were delivered with different schedules (9 and 6 h a day for 2–3 weeks, respectively). The follow-up assessment (4.5 and 6 months follow-up, respectively) was not identical (13, 19), which may have introduced a bias.

Despite the increasing focus on intensive UE treatment, optimal dosing information is not known. It was noted by Sakzewski et al. that 60 h was better than 30 h (42), and by Gordon et al. that 90 h lead to better retention of gains than 60 h (19). However, the optimal dosage is likely to vary for each child, and it may be that 90 h exceeds that amount. Thus, the lack of differences between HABIT and HABIT-ILE might be due to the fact that any attenuated UE improvements due to multitasking for children in the HABIT-ILE group could not be observed as 90 h still result in being above the minimal dosing threshold. Thus comparative studies at lower dosages would be of interest. Moreover, the difference in dosing schedule between both interventions may also have affected the results. A RCT comparing 90 h of the same intervention with 6 h a day for 3 weeks or 9 h a day for 2 weeks should disentangle the potential effect of dosage vs intervention content.

While only small differences in the number of subjects between groups are observed in primary outcomes, larger differences can be found in some of the secondary outcomes such as the JTTHF and the COPM as those tests were not included in the testing battery of the first year of the HABIT-ILE intervention. Although the sample size remains sufficient, these differences in sample size may have underpowered the comparisons.

Finally, because HABIT focuses solely on the UE, no LE assessment was performed before and after HABIT interventions. Thus, it was not possible to test whether some differences observed between both interventions are also present in LE motor function improvements. In the future, it would be of interest to perform some relevant LE assessments during HABIT to compare with the results observed during HABIT-ILE.

In conclusion, this retrospective study demonstrated that changes in the UE of children with USCP undergoing 90 h of intensive bimanual intervention do not differ either with (HABIT-ILE) or without (HABIT) a LE component. In addition to the improved UE motor function, a systematic simultaneous engagement focusing on both UE and LE during intensive intervention in children with USCP (HABIT-ILE) leads to larger improvements in tools measuring functional goals and functional daily living activities where LE, posture, and UE–LE coordination are involved. These results need to be confirmed through RCTs, notably to disentangle effects of dosage vs content of intervention. The present study was solely focused on children with USCP. Future studies should also investigate other patterns of CP such as di-, tri-, or quadriplegia to determine therapeutic interventions allowing to maximize improvements as well as the optimal dosage required for these children.

## Ethics Statement

This study was carried out in accordance with the recommendations and approved by Teachers College, Columbia University, Institutionnal Review Board and Comité d’Ethique Hospitalo-Facultaire, Université catholique de Louvain, Facutlé de Médecine, Cliniques Universitaires Saint-Luc. All subjects gave written informed consent in accordance with the Declaration of Helsinki.

## Author Contributions

GS performed the statistical analyses, conducted the literature search, and drafted the manuscript. GS, AG, and YB contributed to the study design. All authors participated in the data collection, data interpretation, critically revised the draft of the manuscript for important intellectual content, and contributed to the writing. All authors have read and approved the final manuscript with agreement to be accountable for all aspects of the work. Data sharing statement: dataset is available from YB at yannick.bleyenheuft@uclouvain.be.

## Conflict of Interest Statement

The authors declared no potential conflicts of interest with respect to the research, authorship, and/or publication of this article.

## References

[1] Bax M Goldstein M Rosenbaum P Leviton A Paneth N Dan B Proposed definition and classification of cerebral palsy, April 2005. Dev Med Child Neurol (2005) 47(8):571–6. 10.1017/S001216220500112X 16108461

[2] Murphy CC Yeargin-Allsopp M Decoufle P Drews CD. Prevalence of cerebral palsy among ten-year-old children in metropolitan Atlanta, 1985 through 1987. J Pediatr (1993) 123(5):S13–20. 10.1016/S0022-3476(05)80892-3 8229472

[3] Bell KJ Ounpuu S DeLuca PA Romness MJ. Natural progression of gait in children with cerebral palsy. J Pediatr Orthop (2002) 22(5):677–82. 10.1097/00004694-200209000-00020 12198474

[4] Bleyenheuft Y Gordon AM. Precision grip control, sensory impairments and their interactions in children with hemiplegic cerebral palsy: a systematic review. Res Dev Disabil (2013) 34(9):3014–28. 10.1016/j.ridd.2013.05.047 23816634

[5] Novak I McIntyre S Morgan C Campbell L Dark L Morton N A systematic review of interventions for children with cerebral palsy: state of the evidence. Dev Med Child Neurol (2013) 55(10):885–910. 10.1111/dmcn.12246 23962350

[6] Gordon AM Charles J Wolf SL. Methods of constraint-induced movement therapy for children with hemiplegic cerebral palsy: development of a child-friendly intervention for improving upper-extremity function. Arch Phys Med Rehabil (2005) 86(4):837–44. 10.1016/j.apmr.2004.10.008 15827942

[7] Charles J Gordon AM. Development of hand-arm bimanual intensive training (HABIT) for improving bimanual coordination in children with hemiplegic cerebral palsy. Dev Med Child Neurol (2006) 48(11):931–6. 10.1017/S0012162206002039 17044964

[8] Domagalska ME Szopa AJ Lembert DT. A descriptive analysis of abnormal postural patterns in children with hemiplegic cerebral palsy. Med Sci Monit (2011) 17(2):Cr110–6. 10.12659/MSM.881396 21278687 PMC3524706

[9] Prabhu SB Diermayr G Gysin P Gordon AM Coordination of fingertip forces in object transport during gait in children with hemiplegic cerebral palsy. Dev Med Child Neurol (2011) 53(9):865–9. 10.1111/j.1469-8749.2011.04061.x 21790557

[10] Hung YC Meredith GS. Influence of dual task constraints on gait performance and bimanual coordination during walking in children with unilateral cerebral palsy. Res Dev Disabil (2014) 35(4):755–60. 10.1016/j.ridd.2014.01.024 24529863

[11] Eliasson AC Forssberg H Hung YC Gordon AM. Development of hand function and precision grip control in individuals with cerebral palsy: a 13-year follow-up study. Pediatrics (2006) 118(4):e1226–36. 10.1542/peds.2005-2768 17015511

[12] Bleyenheuft Y Gordon AM. Hand-arm bimanual intensive therapy including lower extremities (HABIT-ILE) for children with cerebral palsy. Phys Occup Ther Pediatr (2014) 34(4):390–403. 10.3109/01942638.2014.932884 25271469

[13] Bleyenheuft Y Arnould C Brandao MB Bleyenheuft C Gordon AM. Hand and Arm bimanual intensive therapy including lower extremity (HABIT-ILE) in children with unilateral spastic cerebral palsy: a randomized trial. Neurorehabil Neural Repair (2015) 29(7):645–57. 10.1177/1545968314562109 25527487

[14] Bleyenheuft Y Ebner-Karestinos D Surana B Paradis J Sidiropoulos A Renders A Intensive upper- and lower-extremity training for children with bilateral cerebral palsy: a quasi-randomized trial. Dev Med Child Neurol (2017) 59(6):625–33. 10.1111/dmcn.13379 28133725

[15] Brandao MB Ferre C Kuo HC Rameckers EA Bleyenheuft Y Hung YC Comparison of structured skill and unstructured practice during intensive bimanual training in children with unilateral spastic cerebral palsy. Neurorehabil Neural Repair (2013) 28(5):452–61. 10.1177/1545968313516871 24376067

[16] Eliasson AC Krumlinde-Sundholm L Rosblad B Beckung E Arner M Ohrvall AM The Manual Ability Classification System (MACS) for children with cerebral palsy: scale development and evidence of validity and reliability. Dev Med Child Neurol (2006) 48(7):549–54. 10.1017/S0012162206001162 16780622

[17] Gordon AM Schneider JA Chinnan A Charles JR. Efficacy of a hand-arm bimanual intensive therapy (HABIT) in children with hemiplegic cerebral palsy: a randomized control trial. Dev Med Child Neurol (2007) 49(11):830–8. 10.1111/j.1469-8749.2007.00830.x 17979861

[18] Kleim JA Jones TA. Principles of experience-dependent neural plasticity: implications for rehabilitation after brain damage. J Speech Lang Hear Res (2008) 51(1):S225–39. 10.1044/1092-4388(2008/018) 18230848

[19] Gordon AM Hung YC Brandao M Ferre CL Kuo HC Friel K Bimanual training and constraint-induced movement therapy in children with hemiplegic cerebral palsy: a randomized trial. Neurorehabil Neural Repair (2011) 25(8):692–702. 10.1177/1545968311402508 21700924

[20] Arnould C Penta M Renders A Thonnard JL. ABILHAND-Kids: a measure of manual ability in children with cerebral palsy. Neurology (2004) 63(6):1045–52. 10.1212/01.WNL.0000138423.77640.37 15452296

[21] Krumlinde-sundholm L Eliasson A-C Development of the Assisting Hand Assessment: a Rasch-built measure intended for children with unilateral upper limb impairments. Scand J Occup Ther (2003) 10(1):16–26. 10.1080/11038120310004529

[22] Krumlinde-Sundholm L Holmefur M Kottorp A Eliasson AC. The Assisting Hand Assessment: current evidence of validity, reliability, and responsiveness to change. Dev Med Child Neurol (2007) 49(4):259–64. 10.1111/j.1469-8749.2007.00259.x 17376135

[23] Jebsen RH Taylor N Trieschmann RB Trotter MJ Howard LA An objective and standardized test of hand function. Arch Phys Med Rehabil (1969) 50(6):311–9. 5788487

[24] Taylor N Sand PL Jebsen RH Evaluation of hand function in children. Arch Phys Med Rehabil (1973) 54(3):129–35. 4696054

[25] Reedman SE Beagley S Sakzewski L Boyd RN The Jebsen Taylor Test of hand function: a pilot test-retest reliability study in typically developing children. Phys Occup Ther Pediatr (2015) 36(3):292–304. 10.3109/01942638.2015.1040576 26422369

[26] Beagley SB Reedman SE Sakzewski L Boyd RN. Establishing Australian Norms for the Jebsen Taylor Test of hand function in typically developing children aged five to 10 years: a pilot study. Phys Occup Ther Pediatr (2016) 36(1):88–109. 10.3109/01942638.2015.1040571 26422461

[27] Haley SM Pediatric Evaluation of Disability Inventory (PEDI): Development, Standardization and Administration Manual. Boston: PEDI Research Group (1992).

[28] Haley SM Coster WJ Dumas HM Fragala-Pinkham MA Kramer J Ni P Accuracy and precision of the Pediatric Evaluation of Disability Inventory computer-adaptive tests (PEDI-CAT). Dev Med Child Neurol (2011) 53(12):1100–6. 10.1111/j.1469-8749.2011.04107.x 22077695 PMC3638866

[29] Berg M Jahnsen R Froslie KF Hussain A. Reliability of the Pediatric Evaluation of Disability Inventory (PEDI). Phys Occup Ther Pediatr (2004) 24(3):61–77. 10.1300/J006v24n03_05 15257969

[30] Law M Baptiste S McColl M Opzoomer A Polatajko H Pollock N. The Canadian occupational performance measure: an outcome measure for occupational therapy. Can J Occup Ther (1990) 57(2):82–7. 10.1177/000841749005700207 10104738

[31] Dedding C Cardol M Eyssen IC Dekker J Beelen A. Validity of the Canadian Occupational Performance Measure: a client-centred outcome measurement. Clin Rehabil (2004) 18(6):660–7. 10.1191/0269215504cr746oa 15473118

[32] Verkerk GJ Wolf MJ Louwers AM Meester-Delver A Nollet F. The reproducibility and validity of the Canadian Occupational Performance Measure in parents of children with disabilities. Clin Rehabil (2006) 20(11):980–8. 10.1177/0269215506070703 17065541

[33] Schmider E Ziegler M Danay E Beyer L Bühner M Is it really robust? Methodology (2010) 6:147–51. 10.1027/1614-2241/a000016

[34] Howell D Statistical Methods for Psychology. 7th ed Belmont, CA: Cengage Wadsworth (2007).

[35] Norman GR Sloan JA Wyrwich KW. Interpretation of changes in health-related quality of life: the remarkable universality of half a standard deviation. Med Care (2003) 41(5):582–92. 10.1097/00005650-200305000-00007 12719681

[36] Katz-Leurer M Rotem H Meyer S. Effect of concurrent cognitive tasks on temporo-spatial parameters of gait among children with cerebral palsy and typically developed controls. Dev Neurorehabil (2014) 17(6):363–7. 10.3109/17518423.2013.810676 23924194

[37] Krumlinde-Sundholm L Reporting outcomes of the Assisting Hand Assessment: what scale should be used? Dev Med Child Neurol (2012) 54(9):807–8. 10.1111/j.1469-8749.2012.04361.x 22803624

[38] Sakzewski L Ziviani J Abbott DF Macdonell RA Jackson GD Boyd RN. Randomized trial of constraint-induced movement therapy and bimanual training on activity outcomes for children with congenital hemiplegia. Dev Med Child Neurol (2011) 53(4):313–20. 10.1111/j.1469-8749.2010.03859.x 21401585

[39] Sakzewski L Miller L Ziviani J Abbott DF Rose S Macdonell RA Randomized comparison trial of density and context of upper limb intensive group versus individualized occupational therapy for children with unilateral cerebral palsy. Dev Med Child Neurol (2015) 57(6):539–47. 10.1111/dmcn.12702 25627092

[40] Chiarello LA Palisano RJ Maggs JM Orlin MN Almasri N Kang LJ Family priorities for activity and participation of children and youth with cerebral palsy. Phys Ther (2010) 90(9):1254–64. 10.2522/ptj.20090388 20576716

[41] Ostensjo S Oien I Fallang B Goal-oriented rehabilitation of preschoolers with cerebral palsy – a multi-case study of combined use of the Canadian Occupational Performance Measure (COPM) and the Goal Attainment Scaling (GAS). Dev Neurorehabil (2008) 11(4):252–9. 10.1080/17518420802525500 19031197

[42] Sakzewski L Provan K Ziviani J Boyd RN. Comparison of dosage of intensive upper limb therapy for children with unilateral cerebral palsy: how big should the therapy pill be? Res Dev Disabil (2015) 37:9–16. 10.1016/j.ridd.2014.10.050 25460215

